# Establishment of patient-derived 3D *in vitro* models of sarcomas: literature review and guidelines on behalf of the FORTRESS working group

**DOI:** 10.1016/j.neo.2025.101171

**Published:** 2025-05-03

**Authors:** Lore De Cock, Ieva Palubeckaitė, Francesca Bersani, Tobias Faehling, Sandro Pasquali, Sam Umbaugh, Michael Torsten Meister, Molly R. Danks, Piotr Manasterski, Richard Miallot, Manuela Krumbholz, Siyer Roohani, Dominique Heymann, Florencia Cidre-Aranaz, Agnieszka Wozniak, Patrick Schöffski, Judith V.M.G. Bovée, Alessandra Merlini, Sanne Venneker

**Affiliations:** a Laboratory of Experimental Oncology, KU Leuven, Leuven Cancer Institute, Leuven, Belgium; b Department of General Medical Oncology, University Hospitals Leuven, Leuven Cancer Institute, Leuven, Belgium; c Department of Pathology, Leiden University Medical Center, Leiden, the Netherlands; d Department of Oncology, Translational Oncology Laboratory "Paola Gilardi", University of Turin, Turin, Italy; e Hopp Children’s Cancer Center Heidelberg (KiTZ), Heidelberg, Germany; f National Center for Tumor Diseases (NCT), NCT Heidelberg, a partnership between DKFZ and Heidelberg University Hospital, Heidelberg, Germany; g Division of Translational Pediatric Sarcoma Research, German Cancer Research Center (DKFZ), German Cancer Consortium (DKTK), Heidelberg, Germany; h Faculty of Medicine, Heidelberg University, Heidelberg, Germany; i Molecular Pharmacology, Department of Experimental Oncology, Fondazione IRCCS Istituto Nazionale dei Tumori, Milan, Italy; j Division of Applied Functional Genomics, German Cancer Research Center (DKFZ), Heidelberg, Germany; k Princess Máxima Center for Pediatric Oncology, Utrecht, the Netherlands; l Oncode Institute, Utrecht, the Netherlands; m Cancer Research UK Edinburgh Centre, Institute of Genetics and Cancer, University of Edinburgh, Edinburgh, United Kingdom; n Department of Surgical and Interventional Sciences, McGill University, Montreal, QC, Canada; o Cancer Research Program, The Research Institute of the McGill University Health Centre, Montreal, QC, Canada; p University Hospital Erlangen, Department of Pediatrics Erlangen, Germany; q Charité − Universitätsmedizin Berlin, corporate Member of Freie Universität Berlin and Humboldt-Universität zu Berlin, Department of Radiation Oncology, Berlin, Germany; r Berlin Institute of Health at Charité-Universitätsmedizin Berlin, BIH Biomedical Innovation Academy, BIH Charité (Junior) Clinician Scientist Program, Berlin, Germany; s Nantes Université, CNRS, UMR6286, US2B, Institut de Cancérologie de l'Ouest, Saint-Herblain, France; t Université of Sheffield, School of Medicine and Population Health, Sheffield, United Kingdom; u Division of Medical Oncology, San Luigi Gonzaga University Hospital, Orbassano, Turin, Italy

**Keywords:** 3D *in vitro* models, Tumoroids, Patient-derived preclinical models, Sarcoma

## Abstract

Sarcomas are a large family of rare and heterogeneous mesenchymal tumors, which respond poorly to available systemic treatments. Translation of preclinical findings into clinical applications has been slow, limiting improvements in patients’ outcomes and ultimately highlighting the need for a better understanding of sarcoma biology to develop more effective, subtype-specific therapies. To this end, reliable preclinical models are crucial, but the development of 3D *in vitro* sarcoma models has been lagging behind that of epithelial cancers. This is primarily due to the rarity and heterogeneity of sarcomas, and lack of widespread knowledge regarding the optimal growth conditions of these *in vitro* models. In this review, we provide an overview of currently available sarcoma tumoroid models, together with guidelines and suggestions for model development and characterization, on behalf of the FORTRESS (Forum For Translational Research in Sarcomas) international research working group on 3D sarcoma models.

## Introduction

Sarcomas are a family of rare cancers of mesenchymal origin, accounting for <1 % of all adult cancers, but over 20 % of all pediatric solid malignancies [1]. The latest classification of the World Health Organization (WHO) describes >80 different sarcoma subtypes, which can arise in any anatomical location. In general, they can be divided into two broad subcategories: bone sarcomas and soft tissue sarcomas (STS) [2]. The extensive heterogeneity of sarcomas and the very low incidence of each subtype leads to challenges in diagnosis and treatment. Additionally, most sarcomas are resistant to currently available systemic treatment options, leaving multimodal treatment regimens combining surgery with or without radio- or chemotherapy as the only effective treatment option for the majority of the subtypes. This results in poor survival outcomes, especially in patients with unresectable or metastatic sarcomas, with a reported median overall survival rate of 1-2 years for patients with metastatic STS [3]*.* Notwithstanding the urgent need for advances in sarcoma research, which could ultimately lead to improved patient care and outcomes, the translation of laboratory findings to clinical applications has been slow and inefficient [4,5]. Reliable preclinical models are essential research tools to further understand the biology of the different sarcoma subtypes and lead to development of more effective, subtype-specific treatments. Additionally, the number of sarcoma patients who can be enrolled in clinical trials is limited and as such, it is even more important to evaluate potential anti-cancer drugs in reliable preclinical models to efficiently prioritize them prior to considering clinical studies.

**Figure 1:**
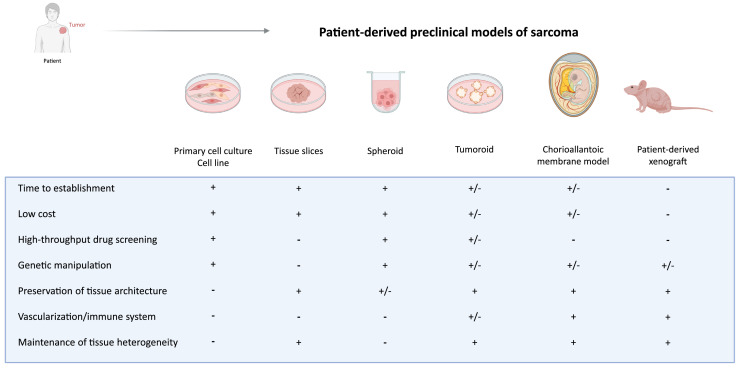
Overview of advantages (+) and disadvantages (-) of the most commonly used patient-derived preclinical sarcoma models. Image was created in BioRender (De Cock, L. (2025) https://BioRender.com/o16p656).

An overview of the advantages and disadvantages of existing patient-derived preclinical models of sarcomas is shown in Figure 1. Two-dimensional (2D) cell lines and primary cell cultures are widely used: these models are in general easy to maintain and manipulate and, as such, very time- and cost-efficient. A recent publication described the sarcoma cell lines reported on Cellosaurus (a resource attempting to describe all cell lines used in biomedical research) [6]. A total of 819 cell lines derived from patients with sarcoma were described, corresponding to 45 sarcoma subtypes as defined by the latest WHO classification. Nevertheless, only a limited number of these cell lines are available through public repositories, of which some are poorly characterized. In addition, an important disadvantage of the use of 2D cell lines and primary cultures is the lack of a three-dimensional (3D) structure and a poor representation of the intra-tumor heterogeneity of the original tumor sample [7]. Moreover, it has been shown that osteosarcoma cell lines are prone to genomic and phenotypic instabilities in long-term cultures [8,9], making them less representative of the clinical situation and which might influence the reproducibility of experiments within and across laboratories using the same cell line. To which extent genomic and phenotypic instability occurs in cell lines of other sarcoma subtypes remains to be explored [10]. Whilst primary cultures are usually short-term, they may have a low tumor cell percentage and a relatively slow growth rate, leading to purity and expansion issues hampering preclinical studies. Together, these factors will have a negative impact on the clinical translation of experimental findings acquired by using 2D preclinical models.

*In vivo* models have also been explored in sarcoma research and one of the most commonly used model is the murine patient-derived xenograft (PDX) model. It is created by implanting tumor fragments from a patient in a (partially) immunodeficient mouse. PDXs represent more complex model systems, better preserving and recapitulating the tissue architecture and heterogeneity of the original tumor than 2D cell lines or primary cell cultures. Notably, PDX tumors can be passaged *in vivo* over time, either as fragments or dissociated cells, thus continuously providing material for downstream *in vitro* experiments or a model for additional *in vivo* studies. The use of PDX models has been invaluable for the sarcoma community and drug testing experiments with PDX models have been able to guide the set-up of subsequent clinical trials and improve the treatment landscape of patients with sarcoma [11,12]. However, the scientific community generally aims to reduce, replace and refine the number of animal experiments as much as possible, in accordance with the 3R principle [13]. Secondly, relatively low establishment rates have been observed, with tumors exhibiting low risk pathologic features being unlikely to be modelled [14]. As such, the number of available sarcoma PDX models is limited, particularly for rare and less aggressive sarcoma subtypes [15]. Additionally, the use of (partially) immunodeficient mice is time- and cost-consuming, as it can take up to 6 months to create a PDX model for slow-growing tumors. An important limitation of the use of (partially) immunodeficient mice is that these do not allow the study of the complex interplay between the immune system and the tumor. Immunodeficient mice co-engrafted with human tumors and immune components (“humanized mice”) can be used to overcome this limitation to some extent [16]. Recently, a few humanized mouse models of sarcoma have been described and used to study different forms of immunotherapy [17,18]. However, only part of the immune cell populations can be effectively restored in humanized mice. Several approaches have been attempted to reproduce human immunity and each of them is characterized by specific limitations [16,19].

To avoid the use of mammalian vertebrate models, non-murine *in vivo* models, such as the chorioallantoic membrane (CAM) model can be used. The CAM is a non-innervated extra embryonal membrane of avian embryos formed under the eggshell during the first third of embryonal development, which presents a well-vascularized, easily accessible platform for transplantation of tumor fragments, dissociated tumor cells or even circulating tumor cells [[20], [21], [22]]. Due to the natural immunodeficiency until the last days of development, there is no need for pharmacological or genetic immunosuppression and most cancer cell lines show high engraftment rates [23]. An additional major advantage of the CAM model over *in vitro* models is that the tumor microenvironment forming in the xenograft includes invasion of stromal cells and active angiogenesis, as well as active interaction between the host embryo and disseminating cancer cells. Taken together, CAM models allow to study a wide variety of biological functions, including angiogenesis, tumor growth and even migration and metastasis of tumor cells [24]. This can be combined with interventions like pharmacological studies, irradiation, genetic modifications or alterations of environmental factors like hypoxia [[25], [26], [27]]. The CAM model has been used successfully with patient-derived or cell-line derived xenografts of different sarcoma subtypes [25,28]. Even though the throughput for drug screening experiments obtainable with the CAM model is much larger than that of any murine model, both remain limited in comparison to traditional 2D cell lines or spheroids. In addition, the available experimental window is limited by the short development time of the embryo of 7-10 days [29]. Alternative non-murine models, such as zebrafish embryos, can be used as well and allow the tracking of tumor development and metastasis by taking advantage of the larvae's transparency [30,31].

To overcome challenges with the abovementioned model systems, 3D *in vitro* models have been developed as a bridge between the traditional 2D *in vitro* cell lines and PDX models. Amongst the existing 3D models, spheroids, organoids and tissue slice cultures are the most commonly used. Tissue slice cultures or tissue explants consist of an *ex vivo* culture of intact tumor slices. Advantages include the rapid establishment using only a limited amount of fresh tissue at a limited cost. Tissue slices can preserve the original tumor architecture, heterogeneity and interaction with the stroma. Additionally, they can be used for drug screening experiments and subsequent immunohistochemical analyses [32]. On the other hand, they have a finite lifespan and are non-renewable [33]. The terms spheroid and organoid are often used interchangeably, but there are important methodological differences. Typically, spheroids are 3D structures formed by cells that aggregate with a spheroid morphology and consist of a homogeneous cell population (*e.g.,* a cell line established in 2D that grows as a spheroid under specific conditions) [34]. The formation of a 3D structure is facilitated by the use of ultra-low attachment plates, seeding the cells as “hanging drops” on the plate or other techniques [35]. In contrast, an organoid is a self-organized 3D tissue that is typically derived from stem cells, but can also be created from progenitor cells, differentiated cells or cancer cells; it recapitulates the key functional, structural and biological complexity of the tissue of origin [36]. As such, tumor organoids (also called tumoroids) are established directly from tumor tissue (straight-from-patient-derived models), without cell expansion in 2D. They represent more complex structures and better reflect tissue heterogeneity as compared to 2D cell cultures and spheroids. Indeed, self-renewal property of tumoroids leads to the growth of different populations sampled from a tumor, offering a better representation of tumor heterogeneity as compared to other tumor models. After tissue dissociation, single cells are either embedded in an extracellular matrix (ECM) (scaffold-based models) or aggregated (scaffold-free models). The scaffold-free method is especially of interest for sarcomas as some histological subtypes produce their own ECM, omitting the need to provide an artificial ECM. Both scaffold-based and scaffold-free tumoroid models (collectively referred to as tumoroids) are usually supplemented with tumor histology-specific media containing essential growth factors, typically without undefined components like fetal bovine serum (FBS) [36].

The organoid technology was originally developed by stimulating stem cell populations within healthy tissue. Sato *et al*. demonstrated that by providing a physiological environment, mouse intestinal stem cells could form a differentiated intestinal epithelium, which was able to proliferate and self-renew [37]. Since then, organoid technology has been applied to various other tissues and translated into the development of organoids representing several diseases, including cancer, which has led to the development of tumoroids [37,38]. These tumoroids can be expanded and used for drug screening, genome editing or functional studies [39]. In addition, co-culture systems can compensate for the lack of vascularization and immune cells. In fact, co-culturing of immune cells with tumoroids to explore tumor ecosystem biology or investigate novel immunomodulating therapeutics has already been successfully implemented in epithelial cancer research [[40], [41], [42]]. The first study on sarcoma tumoroid cultures combined with patient-matched immune cell components (*e.g.,* peripheral blood mononuclear cells or lymph node cells) to examine the effect of immunotherapies was recently published [43]. Techniques to vascularize tumoroids depend on co-culture models with endothelial cells, mesodermal progenitor cells or vascular organoids. Engraftment of the tumoroids in immunodeficient mice models can also induce vascularization [44]. Lastly, the organ-on-a-chip technology can be used to mimic the physiological and mechanical conditions of the human body. These microfluidic cell culture devices consist of an irrigation-controlled microchannel for the growth of living cells and can be combined with different cell types. As such, the microenvironment of organs and their developmental stages can be mimicked and the interaction with tumor cells can be observed [45,46].

Although the tumoroid technology is rapidly growing in the field of epithelial cancers, the transition of this technology to non-epithelial tumors (including sarcomas) has proven difficult for several reasons. First, the rarity and heterogeneity of sarcomas result in a limited availability of samples for establishing preclinical models. Additionally, the cell-of-origin for many sarcoma subtypes is still not known or fully characterized. For colorectal carcinoma, organoid technology was initially developed from healthy epithelial tissue and later translated to the corresponding cancer type. For most sarcoma subtypes, no corresponding normal tissue is available and optimization of the growing conditions has to be performed on tumor samples. These samples are scarcely available, especially when focusing on a single histological subtype, and are often difficult to grow in culture [47]. As compared to epithelial cancers, the biology of sarcomas is poorly understood and the optimal *in vitro* growth conditions are often unknown. The number of publicly available protocols to generate 3D sarcoma tumoroids is limited and there are no repositories collecting the current models. In addition, there is limited commercial and economic interest in developing and optimizing sarcoma tumoroid-specific reagents, scaffolds and cell culture media. This is due to the relatively low number of researchers investigating the topic and to the further fragmentation of the market caused by the numerous histological subtypes of sarcoma, for which histotype-specific technical refinements are most likely required. Due to all these factors, the success rate of establishing sarcoma tumoroids is currently low. However, once established and if fast-growing, these models can be expanded and used relatively fast for additional experiments as compared to PDX models [47].

Within this review, we provide an overview of the methods and straight-from-patient-derived 3D *in vitro* models of sarcomas described in the literature. Additionally, practical advice, based on our own experience, is given to move this challenging field forward.

## Tumoroid models of soft tissue and bone sarcomas described in the literature

Table 1 provides an overview of different 3D *in vitro* models of STS and bone sarcomas, derived directly from patient samples, described in the literature. The overview presents scaffold-based and scaffold-free methods, excluding cell line-derived models or primary cells that were first cultured in 2D and subsequently transferred to 3D.

**Table 1:** Overview of tumoroid models of soft tissue- and bone sarcomas described in the literature.

**Publication**	**Subtype (number of models described)**	**Tumor dissociation**	**Matrix / scaffold-free method**	**Media and growth factors**	**Success rate**	**Methods used to confirm presence of tumor cells**	**Remarks / applications**	**Short-term or longer-term model**
Scaffold-based								

Al Shihabi et al., 2022 [54]	Chordoma (7)	Mechanical dissociation and digestion with collagenase IV (200 U/mL) for 2 h at 37°C, red blood cell lysis	Rings of Matrigel	MammoCult medium with MammoCult supplement (10 %), hydrocortisone (480 ng/mL), and heparin (4 µg/mL)	7/7 (100 %): morphology and immunohistopathology characteristics recapitulated the tumor tissue	H&E and IHC (brachyury, Ki-67, pan-cytokeratin, EMA, and S100)	Drug screening	Short-term
Xu et al., 2023 [66]	Chordoma (5)	Mechanical dissociation and digestion with a tissue pre-treatment kit	Matrigel (growth factor reduced)	Advanced DMEM/F12 with glutamax (1x), B27 (1x), nicotinamide (10 mM), N-acetylcysteine (1.25 mM), HEPES (20 mM), gastrin (10 nM), Wnt3a (100 ng/ mL), noggin (200 ng/mL), R-spondin-1 (250 ng/mL), FGF- 10 (10 ng/mL), EGF (50 ng/mL), A83- 01 (500 nM), and ROCK inhibitor Y-27632 (10 µM)	5/5 (100 %): morphology recapitulated the tumor tissue and molecular alterations were retained	H&E, IHC (brachyury), and WES	Drug treatment	Short-term
Wu et al., 2024 [64,65]	Chordoma (3)	Mechanical dissociation and digestion with collagenase IV (200 U/mL) for 2 h at 37°C	Matrigel	MammoCult medium	Not specified	Flow cytometry (B7-H3 and VEGFR)	Co-culture with CAR-T cells	Short-term
Maloney et al., 2020 [67]	Cutaneous fibrosarcoma (1)	Mechanical dissociation, digestion with collagenase and protease for 2 h on a shaker plate at 37°C, red blood cell lysis, and dead cell removal	Methacrylated collagen type I and Heprasil in 1:3 ratio (bioprinter)	DMEM-High Glucose (4.5 g/L) with FBS (10 %), l-glutamine (1 %), and penicillin/streptomycin (1 %)	Not specified	N/A	Drug treatment started on day 7 after bioprinting	Short-term
Wakamatsu et al., 2022 [52]	Epithelioid sarcoma (2)	Mechanical dissociation and digestion with Liberase TH (50 μg/mL) for 15 min at 37°C	Collagen type I (air-liquid interface)	Advanced DMEM/F12 with HEPES (10 mM), glutamax (1x), penicillin/streptomycin (1x), glutamine (1x), nicotinamide (10 mM), N-acetylcysteine (1 mM), A-83-01 (500 nM), B-27 (1x), hEGF (50 ng/mL), h gastrin I (10 nM), recombinant h noggin (100 ng/mL), recombinant h R-spondin (500 ng/mL), SB-202190, and Afamin/Wnt3a conditioned media (10 %)	2/2 (100 %): cultured for >3 passages	H&E, IHC (pan-keratin, vimentin, CD34, INI-1, and PCNA), and RT-PCR (INI-1)	Biobanking, drug treatment, and xenografting in mice	Short-term
Cao et al., 2022 [68]	Gastro-intestinal stromal tumor (1)	Mechanical and enzymatical dissociation (not specified)	Matrigel	Not specified	Not specified	H&E and IHC (DOG-1)	Drug screening	Short-term
Estrada‐Villaseñor et al., 2021 [69]	Giant cell tumor of bone (1)	Mechanical dissociation and digestion with collagenase (0.3 %) for 2 h at 37°C	Bioprinted polycaprolactone scaffold	DMEM with FBS (10 %) and penicillin/streptomycin (1 %)	Not specified	H&E and IHC (p63, cathepsin K, and RANKL)	Not specified	Short-term
Suzuki et al., 2023 [70]	Giant cell tumor of bone (1)	Mechanical dissociation and digestion with Liberase TH (50 μg/mL) for 15 min at 37°C	Collagen type I-A (air-liquid interface)	Advanced DMEM/F12 with HEPES (10 mM), glutamax (1x), penicillin–streptomycin-glutamine (1x), nicotinamide (10 mM), N-acetylcysteine (1 mM), A-83-01 (500 nM), B-27 (1x), EGF (50 ng/mL), gastrin I (10 nM), noggin (100 ng/mL), R-spondin 1 (500 ng/mL), SB-202190 (10 μM), and Afamin/Wnt3a CM (10 %)	1/1 (100 %): molecular alterations and gene expression patterns were partly retained. Reconstitution after cryopreservation was successful.	WES and RNA sequencing	Biobanking, xenografting in mice, and drug treatment	Short-term
He et al., 2020 [51]	Osteosarcoma (1 primary, 5 lung metastases)	Mechanical dissociation, -/+ digestion with collagenase II (1.5 mg/mL), collagenase IV (500 units/mL), hyaluronidase (200 µg/mL), DNase-I (0.1 mg/mL), dispase II (100 µg/mL) + ROCK inhibitor Y-27632 (10 μM) for 30-60 min at 37°C, -/+ red blood cell lysis	Collagen type I (minced tumor tissues) or Matrigel (single-cell suspensions)	Advanced DMEM/F12 with HEPES (1 mM), glutamax (1x), nicotinamide (10 mM), N-acetylcysteine (1 mM), pen-strep glutamine (1x), A83-01 (0.5 μM), B-27 without vitamin A (1x), EGF (50 ng/mL), gastrin (10 nM), noggin (100 ng/mL), RSPO1 (500 ng/mL), SB-202190 (10 μM), and Wnt3a-conditioned media (50 %)	6/6 (100 %): expansion without morphological changes for >6 months. Reconstitution after cryopreservation was successful.	H&E and IHC (TP53, vimentin, SOX9, and Ki-67)	Biobanking, flow cytometry for T-cell distribution, and drug treatment	Close to longer-term (p7 in >6 months)
Nie et al., 2021 and 2022 [71,72]	Osteosarcoma (24-30)	Mechanical dissociation and digestion with trypsin for 45 min at 37°C	Matrigel	DMEM/F12 with l-glutamine and sodium bicarbonate supplemented with FBS (10 %), penicillin (100 U/mL), streptomycin (100 μg/mL), nystatin (20 μg/mL), nicotinamide (10 mM), N-acetylcysteine (1 mM), A83-01 (0.5 μM), B-27 minus vitamin A (1x), EGF (50 ng/mL), RSPO1 (500 ng/mL), SB-202190 (10 μM), ROCK inhibitor Y-27632 (10 μmol/L), and noggin (100 ng/mL)	30/32 (94 %) from 24 unique tumors: expansion without morphological changes for >3 months. Expression of markers was retained.	H&E and IF (SOX9, vimentin, CD133, and GPC3)	Drug treatment	Short-term
Huang et al., 2024 [73]	Osteosarcoma (not specified)	Mechanical dissociation and digestion with Tumor Tissue Digestion Solution at 37°C for 40 min	Organoid Culture Matrigel	Organoid Culture Media	Not specified	IHC (PDGFD and PDGFRB)	Not specified	Short-term
Zhang et al., 2024 [63]	Osteosarcoma (2)	Mechanical dissociation and digestion with trypsin for 45 min at 37°C	Matrigel	DMEM/F12 with l-glutamine and sodium bicarbonate supplemented with FBS (10 %), penicillin (100 U/mL), streptomycin (100 μg/mL), nystatin (20 μg/mL), nicotinamide (10 mM), N-acetylcysteine (1 mM), A83-01 (0.5 μM), B-27 minus vitamin A (1x), EGF (50 ng/mL), RSPO1 (500 ng/mL), SB-202190 (10 μM), ROCK inhibitor Y-27632 (10 μmol/L), and Noggin (100 ng/mL)	Not specified	N/A	Drug treatment and co-culture with bacteria	Short-term
De Vita et al., 2021 [74]	Rhabdomyosarcoma (1)	Mechanical dissociation and digestion with collagenase type I (2 mg/mL) for 15 min at 37°C, followed by overnight at RT	Collagen type I	DMEM with FBS (10 %), penicillin/streptomycin (1 %), and glutamine (1 %)	1/1 (100 %): morphology recapitulated the tumor tissue and molecular alterations were retained	H&E, IHC (MYOD1), NGS (DNA/RNA), and MSI analysis	Drug treatment	Short-term
Meister et al., 2022 [47]	Rhabdomyosarcoma (19)	Mechanical dissociation, red blood cell lysis buffer if needed	Minced tissue pieces in BME type 2 or single-cell suspensions in BME-supplemented medium.	Advanced DMEM/F12 with penicillin/streptomycin (100 U/mL), glutamax (1 %), B27 minus vitamin A (2 %), N2 (1 %), N-acetylcysteine (1.25 mM), MEM non-essential amino acids (1 %), sodium pyruvate (1 mM), heparin (0.5 U/mL), hEGF (20 ng/mL), hFGF-basic (40 ng/mL), hIGF1 (20 ng/mL), ROCK inhibitor Y-27632 (10 µM), and A-83-01 (5 µM)	19/46 (41 %): expression of specific tumor markers was retained and the culture expansion was sufficient for drug screening	H&E, IHC (desmin, myogenin, and MYOD1), RT-(q)PCR, WGS and RNA sequencing	Biobanking, drug treatment, and genetic modification with CRISPR/Cas9	Longer-term (p40 in >3–6 months)
De Vita et al., 2022 [55]	Giant cell tumor of bone (3), desmoplastic fibroma (1)	Mechanical dissociation and digestion with collagenase type I (2 mg/mL) for 15 min at 37°C, followed by overnight at RT	Collagen type I	DMEM with FBS (10 %), penicillin/streptomycin (1 %), and glutamine (1 %)	4/4 (100 %): morphology recapitulated the tumor tissue (25-70 % tumor cells)	H&E	Drug treatment	Short-term
Goto et al., 2023 [56]	Osteosarcoma (4), Ewing sarcoma (2)	Mechanical dissociation and digestion with Dispersion Enzyme Cocktail EZ for 4 h at 37°C	Collagen	PCM-1 or DMEM F-12 medium	Not specified	N/A	Drug screening	Short-term
Roohani et al., 2023 [58]	Undifferentiated pleomorphic sarcoma (1), pleomorphic liposarcoma (1)	Mechanical dissociation, digestion with collagenase IV, DNaseI, dispase at 37 °C for 60 min, and red blood cell lysis	Matrigel, Collagen type I	Culture medium (not specified) with PDGF-BB and ROCK inhibitor Y-27632 (10 µM)	Not specified	H&E and IHC (vimentin, cytokeratin, S-100, and Ki-67)	Irradiation studies	Short-term
De Vita et al., 2021 [57]	Undifferentiated pleomorphic sarcoma (1), leiomyosarcoma (1), liposarcoma (8)	Mechanical dissociation and digestion with collagenase type I (2 mg/mL) for 15 min at 37°C, followed by overnight at RT	Collagen type I	DMEM with FBS (10 %), penicillin/streptomycin (1 %), and glutamine (1 %)	10/10 (100 %): morphology recapitulated the tumor tissue (10-50 % tumor cells)	H&E	Drug treatment	Short-term
Forsythe et al., 2022 [43]	Angiosarcoma (3), leiomyosarcoma (3), gastrointestinal stromal tumor (1), liposarcoma (1), myxofibrosarcoma (2), dermatofibrosarcoma protuberans (2), pleiomorphic sarcoma (3)	Mechanical dissociation, digestion with collagenase and protease BP at 37°C, and red blood cell lysis	Methacrylated collagen type I and Heprasil	DMEM with FBS (10 %), l-glutamine (1 %), and penicillin/streptomycin (1 %)	16/16 (100 %): definition not specified. Therapeutic testing could be performed in 85 % of cases.	H&E and IHC (Ki-67, PARP1, CD34, MMP9, and vimentin)	Drug treatment started on average on day 7 after seeding culture, co-culture with peripheral blood mononuclear cells or lymph node cells	Short-term
Al Shihabi et al., 2024 [59]	24 sarcoma subtypes	Mechanical dissociation, digestion with collagenase IV, and red blood cell lysis	Rings of Matrigel	MammoCult medium with Mammocult supplement (10 %), hydrocortisone (480 ng/mL), and heparin (4 µg/mL)	114/124 (93 %): morphology recapitulated the tumor tissue, molecular alterations were retained, and culture expansion was sufficient for drug screening	H&E, FISH (liposarcoma: MDM2), and RNA sequencing for a subset of tumoroids	Drug screening	Short-term

Scaffold-free								

Scognamiglio et al., 2019 [75]	Chordoma (not specified)	Digestion with collagenase type-I (1 mg/mL) and trypsin (0.05 %) for 45 min	Matrigel (2 %)-coated microchambers	RPMI with FBS (10 %), penicillin (100 units/mL), and streptomycin (100 μg/mL)	Not specified	IF (CD8 and PD-1)	Drug treatment	Short-term
Bangerter et al., 2023 [48]	Extraskeletal myxoid chondrosarcoma (2)	Mechanical dissociation and digestion with Liberase TM (1 mg/mL) for 4 h	Ultra-low attachment plates	DMEM with horse serum (10 %), glutamax (1x), primocin (100 μg/mL) mixed with CHK Media containing B27 supplement, N-acetylcysteine (1.25 mM), EGF (50 ng/mL), FGF-10 (20 ng/mL), FGF-basic (1 ng/mL), A-83-01 (500 nM), SB202190 (10 μM), nicotinamide (10 mM), PGE2 (1 μM), hydrocortisone (25 nM), epinephrine (0.5 μg/mL) and R-Spondin (conditioned media, self-produced)	2/2 (100 %): morphology recapitulated the tumor tissue and molecular alterations were retained. STR profiling matched the original tumor.	H&E, FISH (NR4A3), NGS, DNA methylation profiling, and STR profiling	Biobanking and drug screening	Longer-term (>p24 in >12 months)
Savary et al., 2023 [49]	Rhabdomyosarcoma (5)	Mechanical dissociation and treatment with collagenase D (0.125 mg/mL)	Ultra-low attachment plates	Low-serum optimized media with penicillin-streptomycin (1x), EGF (10 ng/mL), and basic FGF (1 ng/mL)	5/5 (100 %): definition not specified	H&E, IHC (myogenin and desmin), RNA sequencing, and DNA methylation profiling	Biobanking and drug screening	Longer-term (max. p29)
Zhang et al., 2024 [76]	Osteosarcoma (2)	Mechanical dissociation and digestion with trypsin for 45 min at 37°C	Ultra-low attachment plates	DMEM/F12 with l-glutamine and sodium bicarbonate supplemented with FBS (10 %), penicillin (100 U/mL), streptomycin (100 μg/mL), nystatin (20 μg/mL), nicotinamide (10 mM), N-acetylcysteine (1 mM), A83-01 (0.5 μM), B-27 minus vitamin A (1 ×), EGF (50 ng/mL), RSPO1 (500 ng/mL), SB-202190 (10 μM), ROCK inhibitor Y-27632 (10 μmol/L), and noggin (100 ng/mL)	Not specified	N/A	Drug treatment	Short-term
Boulay et al., 2021 [53]	Synovial sarcoma (4)	Not specified	Ultra-low attachment plates	IMDM with knockout serum replacement (20 %), hEGF (10 ng/mL), basic FGF (10 ng/mL), and penicillin/streptomycin	Not specified	RT-qPCR for *SS18::SSX*	Chromatin analysis and epigenetic studies	Short-term
Gatzweiler et al., 2022 [60]	Malignant rhabdoid tumor (1), embryonal rhabdomyosarcoma (1)	Mechanical dissociation, digestion with trypsin (120 µg/mL) and collagenase II (1 mg/mL) for 60 min at 37°C with repeated mixing every 10 min, DNAse treatment, and red blood cell lysis	Ultra-low attachment plates	Tumor stem medium containing Neurobasal A Medium, DMEM F-12, HEPES Buffer, sodium pyruvate, MEM non-essential amino-acids, l-glutamine, penicillin/streptomycin, B-27, EGF, FGF, PDGF, and heparin	Not specified	DNA methylation profiling and copy number analysis	Drug screening	Short-term
Peterziel et al., 2022 [61]	Malignant rhabdoid tumor (1), Ewing sarcoma (14), osteosarcoma (15), rhabdomyosarcoma (19), sarcoma not otherwise specified (17)	Mechanical dissociation and digestion with trypsin (120 µg/mL) and collagenase II (1mg/mL) for 60 min at 37°C with repeated mixing every 10 min, DNAse treatment, and red blood cell lysis	U-bottom plates	Tumor stem medium containing Neurobasal A Medium, DMEM F-12, HEPES Buffer, sodium pyruvate, MEM non-essential amino acids, l-glutamine, penicillin/streptomycin, B-27, EGF, FGF, PDGF, and heparin	Ranging from 8/19 (42 %, rhabdomyosarcoma) to 1/1 (100 %, malignant rhabdoid tumor): success rate is defined as the possibility to perform a full or partial drug screening experiment	DNA methylation profiling, copy number analysis, and RNA sequencing	Drug screening	Short-term
Chen et al., 2023 [50]	Myxofibrosarcoma (2), malignant peripheral nerve-sheath tumor (1), undifferentiated pleiomorphic sarcoma (1), leiomyosarcoma (1)	Mechanical dissociation and digestion with Liberase TL (0.25 U/mL) for up to 6 hours in a rotator oven at 37°C	Ultra-low attachment plates	Myxofibrosarcoma: Advanced DMEM/F12 with horse serum (10 %), primocin, and glutamax (1x). Others: Advanced DMEM/F12 with glutamax (1x), B27, primocin, N-acetylcysteine (1.25 mM), hEGF (50 ng/mL), hFGF-10 (20 ng/mL), hFGF-basic (1 ng/mL), A-83-01 (500 nM), SB-202190 (10 mM), nicotinamide (10 mM), prostaglandin E2 (1 mM), hydrocortisone (25 nM), epinephrine (0.5 mg/mL), and R-spondin (50 mL, conditioned media, self-produced)	5/10 (50 %): obtained a culture that can be passaged and stably expanded beyond passage 5. Each line underwent a freeze-and-thaw test cycle.	H&E, IHC (leiomyosarcoma: desmin, calponin, and caldesmon; malignant peripheral nerve-sheet tumor: S100 and SOX10), IF (tubulin and vimentin), NGS (DNA/RNA), DNA methylation profiling, and copy number analysis	Biobanking and drug screening	Longer-term (>p30)
Gao et al., 2025 [62]	Undifferentiated pleomorphic sarcoma (39), liposarcoma (34), synovial sarcoma (21), malignant peripheral nerve sheath tumor (15), rhabdomyosarcoma (15), myxofibrosarcoma (15), alveolar soft part sarcoma (11), Ewing sarcoma (10), osteosarcoma (6), epithelioid sarcoma (6), others/unknown (69)	Mechanical dissociation and digestion with collagenase I (200 U/mL) for 1 h	Multi-well plates	Unknown medium with EGF (40 ng/ml), FGF-basic (20 ng/ml), Y-27632-07 (10 µM), HGF (30 ng/ml), GlutaMax (2 nM), penicillin (100 U/ml), B-27 (1x), and MEM non-essential amino acids (1x)	241/254 (95 %, range 86 %−100 %): >500 cell clusters in one week	H&E, IHC, DNA/RNA sequencing, copy number analysis, and single-cell RNA sequencing for a subset of models	Drug screening	Short-term

Abbreviations: CAR: chimeric antigen receptor, DMEM: Dulbecco’s Modified Eagle Medium, DMEM-F12: Dulbecco’s Modified Eagle’s Medium (DMEM) and Ham’s F-12 Nutrient Mixture, DOG-1: discovered on GIST-1, EMA: epithelial membrane antigen, FBS: fetal bovine serum, FISH: fluorescence in situ hybridization, GPC3: glypican 3, H&E: Hematoxylin and eosin, hEGF: human epidermal growth factor, hFGF: human fibroblastic growth factor, hIGF-1: human insulin-like growth factor 1, IHC: immunohistochemistry, IF: immunofluorescence, IMDM: Iscove's Modified Dulbecco's Medium, INI-1: integrase interactor 1, Ki-67: antigen Kiel 67, Liberase-TH: Liberase thermolysin high, Liberase TL: Liberase thermolysin low, Liberase TM: Liberase thermolysin medium, MDM2: murine double minute 2, MEM: minimal essential medium, MMP9: matrix metallopeptidase 9, MSI: microsatellite instability, MYOD1: myogenic differentiation 1, N/A: not applicable, NGS: next-generation sequencing, NR4A3: nuclear receptor subfamily 4 group A member 3, p63: tumor protein P63, PARP1: poly(ADP-ribose) polymerase 1, PCNA: proliferating cell nuclear antigen, PD-1: programmed cell death 1, PDGF(R): platelet-derived growth factor receptor, RT-(q)PCR: (quantitative) reverse transcription polymerase chain reaction, SOX: SRY-box transcription factor, STR: short tandem repeat, TP53: tumor protein P53, VEGFR: vascular endothelial growth factor receptor, WES: whole exome sequencing, WGS: whole genome sequencing.

Models which were only discussed in abstracts are not included. Models are alphabetically ordered based on tumor subtype. Publications including multiple subtypes are mentioned at the bottom of the table.

Several publications mentioned the establishment of only a single culture, while other groups generated a couple of models. However, the success rate of the applied technique to establish the models was often not specified. Additionally, clinical information on the original tumor samples is frequently missing (*e.g.,* primary *versus* metastatic tumor samples or neoadjuvant treatment), whilst the aggressiveness and viability of the tumor cells can heavily influence the success rate of the applied technique and the downstream applications. For instance, Meister et al. reported that the establishment rate differed between fusion-negative and fusion-positive rhabdomyosarcomas (16 % and 83 %, respectively), with the latter being more clinically aggressive. Similarly, the establishment rate was better for relapsed (61 %) compared to primary (30 %) rhabdomyosarcoma tumors [47].

Another term not uniformly specified in the studies listed in Table 1 is whether the established models are short- or longer-term. To classify the models, this review defines longer-term models as sarcoma tumoroids cultured for ≥10 passages and successfully reconstituted after cryopreservation. According to this classification, most established models were short-term and only five out of 29 studies reported on (close to) longer-term models [[47], [48], [49], [50], [51]]. In total, 24 % of the performed studies reported on the biobanking of their 3D models and the successful reconstitution of the cultures after cryopreservation. Four of these studies established longer-term cultures and characterized their models extensively to ensure they were representative of the indicated sarcoma subtype and contained tumor cells, meaning 14 % of the studies provided well-characterized, longer-term sarcoma tumoroid models that can be shared within the sarcoma research community to perform additional preclinical studies [[47], [48], [49], [50]]. This extensive characterization entailed the use of various histopathological and molecular techniques to confirm the histological subtype and the presence of tumor cells, including next-generation sequencing (NGS) approaches to compare the original tumor with the established model and to monitor genetic drift whilst culturing over time. For short-term cultures, such extensive characterization methods are not always feasible due to the limited amount of available material. Less extensive characterization with immunohistochemistry (IHC) or reverse transcription (quantitative) polymerase chain reactions (RT-(q)PCR) for specific tumor markers can already provide sufficient evidence for the presence of tumor cells [[52], [53], [54]].

Confirmation of tumor cells in tumoroids in general is crucial, although not always straightforward to perform due to the lack of specific tumor cell markers in certain sarcoma subtypes (*e.g.,* osteosarcoma). Culture conditions can influence the morphology of cells as well as the expression of certain general markers, meaning hematoxylin and eosin (H&E) staining and/or IHC for general markers should be complemented with the assessment of a marker specific for tumor cells. If such markers are unavailable, NGS-based approaches (*e.g.* low coverage whole genome sequencing to evaluate copy number variations) should be performed on paired samples of the original tumor *versus* the established model to identify genetic alterations that could serve as a specific tumor cell marker. Approximately 50 % of all studies listed in Table 1 did not assess a specific tumor marker as defined by the WHO nor identified a tumor cell marker by using an NGS-based approach to confirm the presence of tumor cells in their established tumoroids. Therefore, these studies should be interpreted with caution until further characterization has been performed. Ideally, the presence of tumor cells needs to be confirmed over several passages; however, this information is often missing in publications. It is also highly recommended to give an indication of the percentage of tumor cells in the tumoroids, either by semi-quantitative scoring of immunohistochemical stains for specific tumor markers or by reporting a variant allele frequency (VAF) of known molecular alterations, but most studies do not elaborate on this.

A wide variety of dissociation methods, media, and scaffolds were used to establish sarcoma tumoroids, suggesting that multiple approaches can be successful, even within one histological sarcoma subtype. Of note, some research groups managed to create models from several sarcoma subtypes with one uniform medium and scaffold [43,50,[55], [56], [57], [58], [59], [60], [61], [62]]. However, most of these models were used for short-term cultures and no information on the possibility of expanding and culturing the models for a longer time was available, raising uncertainty about whether such uniform approaches can also be applied to establish longer-term models. Nevertheless, Chen et al. established tumoroids of four STS subtypes utilizing one consistent approach. Some groups succeeded in co-culturing sarcoma tumoroids with several other cell types, including patient-matched peripheral blood mononuclear cells or cells derived from lymph nodes to test responses to different immune checkpoint inhibitors [43]. Other groups established co-cultures with bacteria or chimeric antigen receptor (CAR)-T cells [[63], [64], [65]].

Table 1 shows that 3D cultures of different sarcoma subtypes were established with various tumor dissociation methods (including both mechanical and enzymatic methods), culture media, and scaffolds, and were subsequently used for several applications. However, definitions of success rate were not consistent, which hampers identifying the most successful method to establish tumoroid models of multiple or single sarcoma subtypes. Moreover, minimal model characterization requirements are not clearly defined within the sarcoma community, which can make it difficult to conclude if the established models are representative of the indicated sarcoma subtype. All studies in which the presence of tumor cells was not confirmed with a specific tumor marker should be interpreted with caution and the models established in these studies should be carefully used for additional applications until further characterization has been performed.

## Methods and challenges for the generation of sarcoma tumoroid models

The most critical steps in generating 3D sarcoma models are the dissociation of the primary tumor sample, the optimization of the 3D model growth conditions, and the cryopreservation for biobanking and subsequent research use.

Different techniques for tumor tissue dissociation can be considered. Crucial steps include: mechanical and/or enzymatic dissociation (often a combination of both techniques is needed), filtering of cell suspensions, red blood cell removal (if necessary), and determination of the cell number and viability (Table 1). To ensure good viability of the isolated tumor cells, tissue should be carefully handled, preserved and processed as soon as possible after the biopsy or surgery, and dissociation time should be minimized to avoid any tissue quality deterioration. The dissociation process and the choice of enzymes might need to be optimized to ensure good viability [77,78]. The success rate improves with the use of fresh tumor tissue and the selection of viable tissue parts within the tumor sample by avoiding necrotic regions. Of note, sarcomas arise in more rigid tissues than epithelial cancers and might need more aggressive dissociation methods to extract the cells from the ECM. Therefore, it is recommended to carefully optimize the dissociation method beforehand and find a proper balance between the number of single cells and cell viability.

The use of 3D scaffolds creates an environment that enhances cell adhesion, proliferation, migration, and cell-cell and cell-matrix interactions. In contrast to epithelial tumoroids, Matrigel or other basement membrane extract (BME) scaffolds, both containing high levels of collagen IV and laminins, will likely not result in optimal growth conditions for all sarcoma tumoroids, as these scaffolds do not mimic all tissue types in which sarcomas can arise (*e.g*., bone sarcomas). Of note, most studies used Matrigel to establish both STS and bone sarcoma scaffold-based tumoroids (Table 1). Matrigel and BME scaffolds could be of interest for sarcomas that are located in ECM characterized by laminins and other basement membrane proteins, such as leiomyosarcoma [79] or lung metastases. The use of other natural-source scaffolds, such as alginate or collagen type 1, can also be considered [80]. The latter is one of the major components of the ECM of most bone sarcomas and would therefore be more suitable. Photocrosslinked natural hydrogels derived from collagen, gelatin and hyaluronic acid are used as bioinks for 3D bioprinting applications and have successfully been used by Forsythe *et al*. and Maloney et al. [43,67]. Other scaffolds, such as methylcellulose, polyethylene glycol, and hydroxyapatite are used in cell line-derived 3D sarcoma models. However, it is unclear if these are also suitable for patient-derived 3D *in vitro* models [81]. Some sarcoma subtypes (*e.g*., chondrosarcoma) can produce their own ECM. It is encouraging to see the production of ECM in the 3D cell culture models, as this cannot be mimicked in the conventional 2D cell culture models [48,82]. Sarcoma cells tend to escape from their surrounding matrix to grow in a monolayer on the culture plastic [47]. Sarcomas arise in tissues with a relatively high stiffness that can exceed the rigidity of standard 2D polystyrene culture plastics (*e.g.,* bone), which could explain their preference for growth as 2D monolayers on plastic surfaces [83]. This can be mitigated by using non-treated or agarose-coated culture plates or by adapting the composition of the 3D matrix, for instance by increasing the stiffness of the gel.

Culture media plays a crucial role in the generation of tumoroids and it is pivotal that a detailed description of the medium is provided to enable other research groups to establish novel sarcoma tumoroid models and reproduce previous results (Table 1). Especially for long-term cultures, optimization of the medium with different growth factors may be subtype specific. This optimization process usually consists of “trial and error” approaches supported by a literature review, which can be challenging, costly and time-consuming. Protocols originally developed for colorectal cancer growth have since been adapted for other epithelial cancers, leading to the successful development of patient-derived tumoroids. However, translating these protocols to sarcomas presents challenges due to the mesenchymal origin of sarcomas and the lack of knowledge regarding the cell of origin for most subtypes. Despite this, the successful generation of patient-derived tumoroids from malignant rhabdoid tumors suggests that protocols for kidney organoid generation may be applied to tumors of a non-epithelial origin arising in the kidney [84,85]. Further research into transcriptomic data to identify active signaling pathways that promote tumor growth may lead to the optimization of culture media for tumoroid growth of specific sarcoma subtypes. For instance, this approach was effective in non-fused rhabdomyosarcoma, where patient-derived tumoroids were established from highly aggressive, relapsing tumors [49]. For short-term cultures, this medium optimization process seems to be less essential and the use of a more uniform medium can be considered (“one size fits all”). The culture media might influence the successful establishment of cultures, however the impact on biological processes should also be critically evaluated. Standard cell culture media do not recapitulate the metabolic environment in patients, with usually higher-than physiologic concentrations of glucose, fat, amino acids, and electrolytes, but lacking trace minerals, vitamins and other components of human fluids [86,87]. Recently, physiological media compositions have been developed that mimic the composition of human plasma. In several carcinomas, it was shown that the media composition affects not only metabolic processes, but also transcriptional pathways related to proliferation and cell motility [[88], [89], [90]]. Additionally, it is recommended to find a reliable and pure source of growth factors to acquire stable and reproducible culture conditions. The effect of the use of different growth factors on the growth conditions of and the biological processes active in sarcoma tumoroids still needs to be investigated in more depth.

Ideally, researchers should be able to biobank tumoroids and preserve them in liquid nitrogen for long-term storage. Before biobanking can be performed, tumoroids are usually first dissociated into single- cell suspensions. This dissociation process should be carefully optimized as it can heavily influence cell viability [78,91]. The composition of the freezing media is another crucial factor in maintaining the viability of tumoroids. The use of different freezing media approaches is described in the literature; common compositions include 50 % culture medium + 40 % FBS + 10 % dimethylsulfoxide (DMSO) or 10 % DMSO in FBS [47]. Commercially available media such as Cryostor CS10 cell freezing medium (Sigma; contains 10 % DMSO, serum-free) and Stem-CellBanker (Ambios, composition not disclosed) or Bambanker medium (Nippon Genetics, composition not disclosed) can be tested as well [47,52]. Viability upon freeze-thaw cycles should be checked to verify the stability of the tumoroid culture and to assess if the established models qualify as long-term models that can be shared with and used by other sarcoma research groups.

Overall, several steps are highly important in establishing viable, growing, and reproducible sarcoma tumoroid models. Each of these individual steps require optimization, potentially even for each sarcoma subtype, and the already established methods from epithelial tumoroids are not always applicable due to differences in the tissue- or cell of origin as compared to sarcomas. Due to the limited availability of patient samples, especially for rare or ultra-rare sarcomas, collaboration within the sarcoma research community is required to identify these optimal culture conditions and the sharing of successful methods, but also non-working methods, will be crucial to achieve this goal.

## Minimal characterization requirements of sarcoma tumoroid models

To ensure the model preserves the characteristics of the original patient sample and to determine the tumor composition of the tumoroid cultures, characterization is needed and recommended, even for short-term cultures if enough material is available. The diagnosis of the original tumor material should be established by an expert sarcoma pathologist to avoid misclassification of the model. Characterization of preclinical models of sarcoma is essential, yet can be very challenging as sarcomas are a heterogeneous group of tumors that can contain different components within one lesion. For instance, certain histological subtypes harbor a well- and dedifferentiated component, such as dedifferentiated liposarcoma and dedifferentiated chondrosarcoma, whilst other sarcoma subtypes contain both an epithelial and spindle cell component (*e.g.,* synovial sarcoma). The characterization of sarcoma tumoroids should not only entail the confirmation of the presence and proportion of tumor cells, but should also include a description of which tumor component(s) are represented in the established model. Furthermore, tumor samples typically harbor non-cancerous cells in the tumor microenvironment (TME), which are often tightly intertwined with tumor cells in the tissue. As noted in epithelial tumoroids, normal cells may easily outgrow cancer cells in culture [92]. While cultured epithelial cells can be more easily distinguished from fibroblasts morphologically, it is sometimes impossible to distinguish cultured fibroblasts from sarcoma cells, given their common mesenchymal phenotype and the lack of specific fibroblast markers.

3D cultures can be collected, fixed and embedded into paraffin to study morphology and expression of sarcoma-specific markers. This ensures that the tumoroid models accurately recapitulate the most relevant histopathological features of the original tumor, and especially the expression of pathognomonic, diagnostic markers (*e.g.,* brachyury expression in chordoma). The 3D cultures are often small and are prone to damage during the embedding process. Histogel (Epredia) or alternatives, such as an agarose coat, might be considered to minimize the risk of altering the sample throughout this delicate procedure [93].

Commonly available, cost-effective techniques with short turn-around-time such as H&E staining and IHC are indeed invaluable to assess whether the 3D model reliably retains similar morphology and marker expression with respect to the specific sarcoma type from which the *in vitro* culture was derived. However, culture conditions can heavily influence cell morphology and the activation of signaling pathways (as described above), underlining the need for additional characterization on the molecular level to confirm the established culture contains tumor cells. It is highly recommended to involve an expert sarcoma pathologist to identify which characterization procedures should be performed to ensure the establishment of a representative model. This can prevent the use of expensive, time-consuming, or even aspecific/unnecessary techniques when addressing histopathological correspondence between the patient sample and the *in vitro* 3D model. Furthermore, early involvement of a pathologist is key to accurately select pathognomonic, specific markers to evaluate similarities between the original patient sample and the derived model. To this purpose, it is essential to differentiate between histopathological model characterization, which requires evaluation by an expert pathologist and determines whether the 3D culture recapitulates the defining, diagnostic features of the sarcoma subtype of origin, and any further model characterization required for research purposes. Although both are fundamental in the process of accurate model characterization and description, concordant preservation of morphological features and diagnostic markers should be the prerequisite for any additional subsequent molecular analysis. Best techniques for the assessment of pathognomonic markers should follow best practice according to the pathologist’s judgement and according to standard procedures for the diagnosis of each specific sarcoma subtype and may encompass additional analyses beyond IHC according to the diagnostic molecular alterations to be identified. For instance, while morphological and IHC evaluation might be sufficient to establish the concordance between a putative 3D model and the original patient sample for chordoma cases using brachyury IHC assessment, fluorescent *in situ* hybridization (FISH) would be required to confirm the presence of a *YAP1-TFE3* rearrangement in a case of non-*CAMTA1*-rearranged epithelioid hemangioendothelioma (EHE), both in the patient’s original sample and in the putative EHE 3D culture. RT-PCR techniques can be used as an alternative for FISH and can be performed early during culturing as it requires a limited number of cells and is highly specific. For certain histological subtypes specific tumor cell markers are not available (*e.g.,* osteosarcoma), meaning an NGS-based paired comparison between the original tumor and the established model should be performed to identify genetic alterations (*e.g.,* mutations, translocations, or copy number alterations) that can serve as a tumor cell marker.

Indeed, as many sarcomas are characterized by genomic alterations, ranging from translocations to point mutations or deletions/insertions/amplifications, it is crucial to confirm the presence of these genetic alterations in the corresponding tumoroids. This can be done by several techniques, such as RT-(q)PCR, Sanger sequencing, NGS, FISH, or IHC depending on the genetic alteration that characterizes the specific sarcoma under investigation. Recently, a few of these genetic hallmarks, previously only evaluable with sequencing or FISH techniques, have been efficiently confirmed in the diagnostic setting by IHC with highly specific antibodies, as in the case of histone H3.3 (H3F3A) p.G34W in giant cell tumor of bone or SSX-SS18 in synovial sarcoma [[94], [95], [96], [97]]. This allows for a more rapid and less expensive marker evaluation, and could be easily applied to *in vitro* model validation in these histological subtypes. However, this is not yet the case for all diagnostic hallmarks for each specific sarcoma type. For instance, FISH remains the preferred method to assess *TFE3* rearrangements, as TFE3 IHC lacks satisfactory specificity and sensitivity for the diagnosis [98]. Another example is the assessment of MYC amplifications in angiosarcoma. It has been shown that MYC expression can be strongly upregulated in culture, which can be a pitfall when using IHC to assess the presence of a MYC amplification in tumoroids [99]. As such, it is recommended to assess the presence of a MYC amplification also on the genetic level, for instance with FISH. In general, translocation-driven sarcomas are still diagnosed by either FISH or RT-PCR techniques. Hence, it is evident that all these considerations are best integrated in 3D model characterization with the upfront involvement of an expert sarcoma pathologist, especially in the rapidly-evolving scenario of diagnostic optimization and efficacy improvements in the field of sarcoma *in vitro* diagnostics.

After assessing the representativeness of the model on the histopathological and molecular level, further model investigation with *multi-omic* approaches can follow for more in-depth model characterization and validation. However, since 3D sarcoma models contain higher cellular heterogeneity, bulk omics methods may not be detailed enough for the investigation of transcriptomic and proteomic changes between the original tumor and the established model. In this case, single-cell omics, like single-cell RNA sequencing, may provide valuable insights into the different cell subtypes present in the 3D culture [47,100,101]. Furthermore, especially for organoids consisting of different cell types or 3D cultures with complex structures, the spatial organization of the different cell types and their interaction within themselves and their microenvironment may be of interest. For this, spatial transcriptomics/proteomics technologies or multiplexed immunofluorescence may be employed [102]. The advent of spatial biology techniques and single-cell analysis techniques, on top of bulk genomic, transcriptomic, proteomic and metabolomic studies, which are invaluable for model description refinement, represents a unique opportunity to dissect and describe the established 3D cultures at multiple levels. Moreover, such *multi-omic* approaches can also aid in enhancing our understanding of tumor biology and the identification of potential treatment options, which can lay a strong foundation for novel hypotheses and the initiation of research projects making use of the established models.

Any contaminations with other cell types should be detected at an early stage to avoid the overgrowth of non-tumor cells or propagation of a culture not representative of the original tumor [47]. Quantification of genetic variants on the DNA level (*e.g.,* the VAF of a known mutation) or semi-quantitative scoring of IHC stains for specific tumor cell markers can be used to quantify the amount of tumor *versus* non-tumor cells. At early passages the available material is limited, influencing the range of assays that can be applied to characterize the established culture. Kits specifically designed to isolate material from a limited number of cells (*e.g.*, RN/DNeasy micro kit from Qiagen) or simultaneously isolate both DNA and RNA from the same sample (*e.g.* Quick-DNA/RNA Miniprep kit from Zymo Research or AllPrep DNA/RNA/Protein Mini Kit from Qiagen) can be useful to overcome some of these problems.

It is recommended to check the tumoroid models on the representativeness of and similarity to the original tumor tissue at different 3D-cell culture passages. This ensures model reliability over time and will identify any clonal selection or genetic drift which will have an impact on tumor biology and might alter the response to experimental treatments. It is advisable to check the molecular and phenotypic correspondence between the original patient sample and the 3D culture already at an early passage, to ensure the culture conditions are not causing selection of TME cells or altering the original tumor cell phenotype in terms of morphology and expression of diagnostic markers. Subsequently, the correspondence between the original tumor sample and the 3D culture should be checked after the culture is established and expanded over a longer period of time, after the thawing of cryopreserved samples, and at the end of the planned experimental window.

Additionally, following good laboratory practice, it is advisable to perform short tandem repeat (STR) profiling to authenticate the tumoroid cultures and confirm their similarity to the original tumor material. This is especially important when multiple tumoroid cultures of the same sarcoma subtype from different patients are established simultaneously, to avoid and/or identify early contamination or a mix-up of the samples.

In summary, a combination of morphological and immunohistochemical markers, as defined by an expert sarcoma pathologist, and any sarcoma subtype-specific molecular alteration (*e.g*., translocation, mutation, or copy number alteration) should be checked regularly during establishment, expansion, and reconstitution of cultures to assure representative 3D sarcoma models are established. If specific tumor markers are unavailable, a paired NGS-based comparison between the original tumor and the established model should be performed to identify genetic alterations that can function as such a tumor cell marker. *Multi-omic* approaches performed on paired samples of the original tumor and established 3D model can be used for further, more in-depth characterization and validation of the established tumoroid models.

## Applications of sarcoma tumoroid models

If the model is thoroughly characterized and recapitulates sarcoma biology, it can be used as a reliable preclinical model for further applications. The definition of a successfully established model is debatable (Table 1), but overall it is agreed that it should be well-characterized, preserving the morphological and molecular characteristics of the original tumor as described above. Additionally, the model should be useful to answer specific research questions of interest. Model characteristics such as the expansion and proliferation rate, but also whether the model is short-term (<10 passages) or longer-term (≥10 passages, including cryopreservation), could be key determinants to define the usefulness and successfulness of a model for individual research studies.

Tumoroid models are frequently utilized for drug screening experiments. These experiments can be performed on short-term cultures (a few days after initiating the culture from primary material), whereby growth conditions are usually not optimized for specific sarcoma subtypes, making them more uniform and less complicated. However, due to a lack of expansion of the culture, usually a limited number of drugs at few concentrations can be tested. Alternatively, tumoroids can be cultured for longer to facilitate expansion and cryopreservation for larger drug screening experiments or future follow-up studies. Nevertheless, longer-term sarcoma tumoroid models remain scarce. Failed attempts of research groups are not published and the models that did succeed have lower and more variably success rates as compared to short-term cultures (Table 1). This is likely explained by the need for a more tailored culture environment (both scaffold and medium) and the need for optimized protocols for subculturing and cryopreservation. The use of bioprinters and/or automated liquid handling devices can be helpful in the successful establishment of culture systems by limiting inconsistencies, improving the ability to scale up, and increasing the throughput of 3D drug screening [67].

Different assays can be used to evaluate cell viability and the half maximal inhibitory concentration (IC_50_) after treatment of 3D cultures, such as CellTiter-Glo 3D (Promega) or PrestoBlue (Invitrogen) [103]. The latter has the advantage that cells can be cultured further after readout as the assay is non-toxic and does not require lysis of cells. IHC for markers of cell proliferation (*e.g.,* antigen Kiel 67 (Ki-67)) and apoptosis (*e.g*., cleaved caspase 3) can also be used to assess treatment efficacy in 3D cultures and to ensure that the 3D culture is still actively dividing and not just cytostatic. In addition, the treatment effect on downstream signaling pathways can be evaluated if specific markers are available. H&E staining can be performed to visualize treatment-induced morphological changes and, in combination with the open source QuPath software (https://qupath.github.io/), to quantify the number of tumoroids within a standardized area or to measure the average tumoroid size after treatment [82,104]. Alternatively, flow cytometry for cell cycle analysis and expression of apoptotic markers or other proteins of interest could be employed, although a high number of cells is needed for this experimental approach which could be a limiting factor. Gene expression changes upon treatment might be assessed by RT-(q)PCR or NGS-based approaches, including single cell- and bulk RNA sequencing.

A more advanced method to measure treatment efficacy in 3D cell cultures are high-content imaging approaches that use fluorescent imaging probes (*e.g.,* for viability, hypoxia, and apoptosis) and/or brightfield imaging to analyze phenotypic features of the tumoroids (*e.g.,* shape, size, volume, and morphology) [105,106]. High-content imaging approaches provide multiple parameters that could be informative to measure treatment response and therefore go beyond the standard cell viability assays usually utilized for drug screens in 3D cell cultures. However, high-content imaging requires advanced microscopes and data analysis pipelines which might not be available in all laboratories, making the use of cell viability assays in combination with morphological or immunohistochemical assessment a more straightforward and feasible approach to study treatment response in 3D cultures.

Both short- and longer-term 3D cell culture models can also be used to study radiosensitivity to photon or proton therapy [58]. The efficacy of radiotherapy can be measured with cell viability assays, quantification of the number of tumoroids within a standardized area, or by applying any of the other readouts that are used for drug response assays. Finally, relatively fast-growing, longer-term 3D cultures can be genetically modified by transducing the tumoroids with expression vectors carrying genes of interest and/or reporter genes, or by applying gene editing approaches, such as CRISPR-Cas9 [107]. These genetically modified derivates can subsequently be used to perform functional studies to elucidate how genetic alterations frequently observed in certain sarcoma subtypes (*e.g., IDH1/IDH2* mutations in chondrosarcoma, *H3F3A* mutations in giant cell tumor of bone, and *SSX::SS18* fusions in synovial sarcoma) drive tumorigenesis, which could aid in the identification of novel targeted therapies. Moreover, the genetically modified 3D cultures, together with their parental counterparts, could be used in drug screening approaches to identify treatments that specifically target cells harboring the genetic alteration of interest (*e.g.,* synthetic lethal interactions).

In conclusion, well-characterized sarcoma tumoroid models can be used for multiple applications, including drug and radiotherapy sensitivity studies as well as genetic modifications. Depending on the research question, both short- and longer-term models might be useful, although certain applications are only feasible when a relatively fast growing, longer-term model is available (*e.g.,* genetic modifications for functional studies). Before using their successfully established 3D cell culture models, researchers should carefully assess if their models are suitable for their intended application and, if not, determine if further optimization of culture conditions (*e.g.,* scaffold and medium) might facilitate this. The use of more advanced *in vitro* sarcoma models, such as sarcoma tumoroids, could aid in enhancing our understanding of sarcoma biology and treatment responses, which can ultimately improve the outcome of patients with sarcoma.

## Conclusion and future perspectives

**Recommendations for the establishment and use of sarcoma tumoroid models**

**Generation**

• The scaffold and culture medium, as well as the tumor dissociation, subculturing, and biobank method, are crucial steps to establish tumoroids and should be optimized for each individual sarcoma subtype based on available literature and the known biological features of the sarcoma subtype of interest.
• As sarcomas are rare cancers with limited availability of tissue specimens, sarcoma research groups are highly encouraged to collaborate and share detailed methods, preliminary experiences, and failed attempts to identify optimal growth conditions to move the field forward more quickly.

**Characterization**

• An expert sarcoma pathologist should be consulted upfront to prevent misclassification and insufficient characterization of the established tumoroids.
• Minimal characterization requirements entail the confirmation of presence of tumor cells in the tumoroid by assessing a tumor cell marker as defined by the WHO classification. If such markers are unavailable, it is recommended to perform a NGS-based paired comparison between the original tissue *versus* the established tumoroid.
• The presence of tumor cells should be confirmed during the establishment, expansion, and reconstitution of tumoroids.
• If enough material is available, extensive characterization of tumoroids on both the histopathological and molecular level, including cell morphology, presence of different characteristic cell populations, tumor cell percentage, additional molecular alterations, and signaling pathway activation, as well as a pairwise comparison between the original tissue *versus* the established tumoroid are highly recommended.

**Application**

• The presence of tumor cells in the tumoroids should be confirmed at the start and end of the experimental window.
• Researchers should assess upfront if their established tumoroids are suitable for the intended application and/or to answer their specific research question.

Alt-text: Unlabelled box

So far, the experience with tumoroids derived from sarcoma tissue is limited and far behind compared to the epithelial tumoroids field. Apart from model establishment, a robust characterization and quality control of the tumoroid cultures is needed to ensure reliability and the correct use of these models, which can ultimately lead to the translation of findings into daily clinical practice. Researchers working on sarcoma must develop a common framework for the minimum requirements that define reliable models. We recommend that researchers characterize their models from the very first passages to the end of the planned experiments in close collaboration with expert sarcoma pathologists, to guarantee correspondence of histopathological and molecular disease hallmarks between the 3D model and the original tumor samples. Additional molecular characterizations, preferably with multiple techniques and multi-level -*omic* approaches, are instrumental to further substantiate similarities and closeness to original patient samples and expand our knowledge of the disease.

Beyond the definition of recommendations and general criteria for *bona fide* model validation, it is important to establish better suited scaffolds and growth media for sarcoma tumoroids. Most sarcoma tumoroid studies use the scaffold and growth medium required for epithelial organoid cultures (Table 1), which does not recapitulate the tissues in which sarcomas arise. So far, no studies have investigated the effect of the scaffold and different growth factors added to the culture medium on the growth rates and behavior of tumoroids generated from different sarcoma subtypes. This can be explained by the limited availability of tissue specimens and the rarity of these tumor types. Additionally, resection specimens are often pretreated with chemo- or radiotherapy, limiting the amount of viable tissue available. These aspects, on top of intrinsic tumor heterogeneity, further complicate the comparison of multiple scaffolds and growth media simultaneously. Therefore, a collaborative effort focusing on understanding which scaffolds and growth factors are essential for optimized growth conditions of sarcoma tumoroids is required, especially for long-term cultures where growth conditions may be subtype-dependent. A recent study performed an in-depth proteomic characterization of the ECM of different subtypes of STS, which could provide an informative starting point to optimize the scaffold for STS tumoroid models [79]. This study decellularized fresh-frozen tumor samples to extract the patient-derived ECM, which was used to study the migration of leiomyosarcoma cell lines. The use of matched patient-derived ECM as a scaffold for growing sarcoma tumoroid models should be explored in future studies. Concerning the optimized selection of cell culture medium, it is recommended to start refining the culture conditions based on components described in the literature; however, these are only available for a few subtypes. Extended optimization of the culture conditions can be very costly and time-consuming and might require advanced technical expertise. Therefore, we encourage researchers to publish detailed methods and to share unpublished protocols and expertise with the sarcoma research community.

Besides taking advantage of unique opportunities represented by international research meetings dedicated to sarcomas, such as the Forum for Translational Research in Sarcomas (FORTRESS), which guarantee independent, critical and collaborative sharing of technical procedures, advanced methods and results, it is crucial that scientists advocate for greater inclusion of negative results in the scientific literature. This is especially important in high failure-risk areas of sarcoma research such as 3D culture establishment and characterization. Even when the scientific aim and the methods are designed and developed according to best laboratory practice, or when a specific attempt was highly successful, it would still be of value to also share and communicate the failed attempts and negative results (*i.e.,* in this context, describing unsuccessful methods for 3D sarcoma cultures) in literature as this saves time, money, and resources within the sarcoma research community. It is advisable that researchers involved in the field lobby relevant stakeholders, to highlight the relevance of this underestimated issue which is currently hampering technical advancements in the field of 3D sarcoma cultures.

Both short- and long-term, well-characterized sarcoma tumoroid models are useful for the sarcoma research community. Very recently, Al Shihabi et al. described the creation of short-term patient-derived tumoroids from >100 sarcoma patients, representing 24 different subtypes using a uniform approach. A well-designed, high-throughput drug screening pipeline made it possible to expose the tumoroids to multiple drug compounds (alone or in combination). Results could be assessed within one week after surgery, meaning the results can potentially inform clinical decision- making. The tumoroid models retained key aspects of tumor heterogeneity and preserved the main histopathological and molecular features of the tumor of origin. However, details on characterization were not shown in-depth for all different sarcoma subtypes. This study highlights the potential to test drugs with patient-derived tumoroids to help predict patients’ response to treatment with the ultimate goal of improving outcome [59]. Patients with irresectable or metastatic disease potentially benefit the most from such personalized drug screening approaches. However, tissue specimens from this category of patients are usually not available or very small (*i.e.,* only biopsies are taken, which yield far less material compared to surgical specimens), which hampers the seeding of enough material to perform drug-screening experiments. Therefore, the establishment of expandable, longer-term models would have some unique strengths, especially when limited starting material is available. The establishment of longer-term models might generate enough cells for drug screening experiments and might allow the study of longer-term treatment responses and/or the development of resistance, which could inform additional *in vivo* experiments and subsequent clinical trials [108,109]. Moreover, cryopreserved longer-term cultures can be shared within the sarcoma research community to perform additional studies, which are needed to improve our understanding of sarcoma biology and to identify effective treatment options.

As we strive to reduce animal experiments according to the 3R principle (replacement, reduction and refinement), the use of alternative models is becoming increasingly important. The role of 3D *in vitro* models is complementary to other preclinical models (Figure 1) and an important intermediate between cell lines and animal models. Validation of results in various preclinical models may enhance the translation of preclinical findings into the clinic. While the establishment of sarcoma tumoroids is challenging, it is feasible and holds significant translational potential. Further research should focus on identifying the optimal culture conditions for sarcoma tumoroid models and we should foster collaboration to exchange expertise, protocols and samples. International, independent research networks such as FORTRESS, which nurtured the birth and development of the 3D sarcoma models working group initiative, are the ideal scientific environment to promote collaboration in this field, which is characterized by challenging, high-risk features, yet bearing impactful potential and translational relevance to reshape and refine the framework of *in vitro* modeling for sarcoma research.

## Funding

Ieva Palubeckaitė, Judith V.M.G. Bovée, and Sanne Venneker were financially supported by the Dutch Research Council (NWO, ZON-MW VICI 170.055 to Judith V.M.G. Bovée). Lore De Cock was supported by the donation of a generous gift from a private donor. Tobias Faehling was supported by the German Academic Scholarship Foundation and the Heinrich F.C. Behr foundation. Sandro Pasquali was supported by AIRC Individual Grant - Next Gen Clinician Scientist “Fondazione 13 marzo” [ID#28546]. Molly R. Danks and Piotr Manasterski were supported by funding from Chief Scientist Office [TCS/22/12] and Sarcoma UK [SUK06.2020]. Richard Miallot was supported by the Leiomyosarcoma Direct Research Foundation, The Desmoid Tumor Research Foundation, and The Desmoid Tumour Foundation of Canada. Siyer Roohani is a participant in the BIH Charité Junior Clinician Scientist Program funded by the Charité – Universitätsmedizin Berlin, and the Berlin Institute of Health (BIH). The research team of Florencia Cidre-Aranaz was supported by the German Cancer Aid (DHK-70114111), and the Dr. Rolf M. Schwiete Stiftung (2020-028 and 2022-31). Alessandra Merlini was supported by PON 2014-2020, DM 1062/2021, PNR 2021-2027, and Desmoid Foundation - Associazione Italiana Tumore Desmoide.

## List of abbreviations

**Table:** 

2D	two-dimensional
3D	three-dimensional
BME	basement membrane extract
CAM	chorioallantoic membrane
CAR	chimeric antigen receptor
CRISPR-Cas9	clustered regularly interspaced short palindromic repeats-CRISPR-associated protein 9
DNA	deoxyribonucleic acid
DMEM	Dulbecco's Modified Eagle's medium
DMEM-F12	Dulbecco's Modified Eagle's medium and Ham's F-12 Nutrient Mixture
DMSO	Dimethyl sulfoxide
DOG-1	discovered on GIST-1
ECM	extracellular matrix
EHE	epithelioid hemangioendothelioma
EMA	epithelial membrane antigen
FBS	fetal bovine serum
FISH	fluorescence *in situ* hybridization
GPC3	glypican 3
H3F3A	histone H3.3
H&E	hematoxylin and eosin
hEGF	human epidermal growth factor
hFGF	human fibroblastic growth factor
hIGF-1	human insulin-like growth factor
IC_50_	half maximal inhibitory concentration
IHC	immunohistochemistry
IF	immunofluorescence
IMDM	Iscove's Modified Dulbecco's medium
INI-1	integrase interactor 1
Ki-67	antigen Kiel 67
Liberase-TH	Liberase thermolysin high
Liberase-TL	Liberase thermolysin low
Liberase-TM	Liberase thermolysin medium
MDM2	murine double minute 2
MEM	minimal essential medium
MMP9	matrix metallopeptidase 9
MSI	microsatellite instability
MYOD1	myogenic differentiation 1
N/A	not applicable
NGS	next-generation sequencing
NR4A3	nuclear receptor subfamily 4 group A member 3
p63	tumor protein P63
PARP1	poly(ADP-ribose) polymerase 1
PCNA	proliferating cell nuclear antigen
PD-1	programmed cell death 1
PDGF(R)	platelet-derived growth factor (receptor)
PDX	patient-derived xenograft
RNA	ribonucleic acid
RT-(q)PCR	reverse transcription (quantitative) polymerase chain reaction
SOX	SRY-box transcription factor
STR	short tandem repeat
STS	soft tissue sarcomas
TME	tumor microenvironment
TP53	tumor protein P53
VAF	variant allele frequency
VEGFR	vascular endothelial growth factor receptor
WES	whole exome sequencing
WGS	whole genome sequencing
WHO	World Health Organization

## CRediT authorship contribution statement

**Lore De Cock:** Conceptualization, Methodology, Visualization, Writing – original draft, Writing – review & editing. **Ieva Palubeckaitė:** Conceptualization, Methodology, Writing – original draft, Writing – review & editing. **Francesca Bersani:** Methodology, Writing – original draft, Writing – review & editing. **Tobias Faehling:** Methodology, Writing – original draft, Writing – review & editing. **Sandro Pasquali:** Methodology, Writing – original draft, Writing – review & editing. **Sam Umbaugh:** Methodology, Writing – original draft, Writing – review & editing. **Michael Torsten Meister:** Methodology, Writing – review & editing. **Molly R. Danks:** Writing – review & editing. **Piotr Manasterski:** Writing – review & editing. **Richard Miallot:** Methodology, Writing – review & editing. **Manuela Krumbholz:** Writing – review & editing. **Siyer Roohani:** Writing – review & editing. **Dominique Heymann:** Methodology, Writing – review & editing. **Florencia Cidre-Aranaz:** Methodology, Supervision, Writing – original draft, Writing – review & editing. **Agnieszka Wozniak:** Funding acquisition, Methodology, Supervision, Writing – review & editing. **Patrick Schöffski:** Funding acquisition, Methodology, Supervision, Writing – review & editing. **Judith V.M.G. Bovée:** Conceptualization, Funding acquisition, Methodology, Supervision, Writing – original draft, Writing – review & editing. **Alessandra Merlini:** Conceptualization, Methodology, Supervision, Writing – original draft, Writing – review & editing. **Sanne Venneker:** Conceptualization, Methodology, Supervision, Writing – original draft, Writing – review & editing.

## Declaration of competing interest

The authors declare that they have no known competing financial interests or personal relationships that could have appeared to influence the work reported in this paper.
